# A Spatially Explicit Metapopulation Model and Cattle Trade Analysis Suggests Key Determinants for the Recurrent Circulation of Rift Valley Fever Virus in a Pilot Area of Madagascar Highlands

**DOI:** 10.1371/journal.pntd.0003346

**Published:** 2014-12-04

**Authors:** Gaëlle Nicolas, Véronique Chevalier, Luciano Michaël Tantely, Didier Fontenille, Benoît Durand

**Affiliations:** 1 International Centre of Research in Agronomy for Development (CIRAD), ES Department– AGIRs Unit, Montpellier, France; 2 Paris-Est University, Anses, Laboratory for Animal Health, Epidemiology Unit, Maisons-Alfort, France; 3 Pasteur Institute of Madagascar, Antananarivo, Madagascar; 4 Research Institute for Development (IRD), MIVEGEC, Montpellier, France; Centers for Disease Control and Prevention, United States of America

## Abstract

Rift Valley fever (RVF) is a vector-borne zoonotic disease that causes high morbidity and mortality in ruminants. In 2008–2009, a RVF outbreak affected the whole Madagascar island, including the Anjozorobe district located in Madagascar highlands. An entomological survey showed the absence of *Aedes* among the potential RVF virus (RVFV) vector species identified in this area, and an overall low abundance of mosquitoes due to unfavorable climatic conditions during winter. No serological nor virological sign of infection was observed in wild terrestrial mammals of the area, suggesting an absence of wild RVF virus (RVFV) reservoir. However, a three years serological and virological follow-up in cattle showed a recurrent RVFV circulation. The objective of this study was to understand the key determinants of this unexpected recurrent transmission. To achieve this goal, a spatial deterministic discrete-time metapopulation model combined with cattle trade network was designed and parameterized to reproduce the local conditions using observational data collected in the area. Three scenarios that could explain the RVFV recurrent circulation in the area were analyzed: (i) RVFV overwintering thanks to a direct transmission between cattle when viraemic cows calve, vectors being absent during the winter, (ii) a low level vector-based circulation during winter thanks to a residual vector population, without direct transmission between cattle, (iii) combination of both above mentioned mechanisms. Multi-model inference methods resulted in a model incorporating both a low level RVFV winter vector-borne transmission and a direct transmission between animals when viraemic cows calve. Predictions satisfactorily reproduced field observations, 84% of cattle infections being attributed to vector-borne transmission, and 16% to direct transmission. These results appeared robust according to the sensitivity analysis. Interweaving between agricultural works in rice fields, seasonality of vector proliferation, and cattle exchange practices could be a key element for understanding RVFV circulation in this area of Madagascar highlands.

## Author Summary

Rift Valley fever (RVF) is a mosquito-borne zoonosis affecting domestic ruminants and humans. The RVF virus (RVFV) may be transmitted either by mosquitoes or through direct contact with body fluids or products from viremic animals. Until now Rift Valley fever (RVF) epidemiological cycle had been described in tropical, hot irrigated or arid areas. In 2008, and for the first time, human and bovine cases were detected in a temperate ecosystem in Madagascar. Based on a large and original set of field data relating to various areas of expertise—serological, virological, socio-economic, environmental and entomological data—we proposed a spatialized meta-population model integrating cattle exchange practices and virus transmission dynamic to test several scenarios that could explain the recurrent circulation of the virus in this ecosystem. This is the first model aiming at simulating RVF transmission in a temperate area and using field data.

## Introduction

Rift Valley fever (RVF) is a mosquito-borne zoonosis of livestock known to be endemic in the African mainland [1]. The virus, member of the *Phlebovirus* genus (*Bunyaviridae* family) is transmitted between ruminants by *Aedes*, *Culex* and *Mansonia* mosquitoes [2], [3]. Recent serological and virological results suggest that it could also be transmitted from ruminant to ruminant by direct contacts with viremic fluids or tissues such as blood or abortion products [4]. The infection causes a severe disease in domestic ruminants (e.g. sheep, goat, cattle), including high mortality rates in young animals and abortion storms in pregnant females [5]. In human, clinical signs usually consist in an influenza-like illness, but severe complications such as encephalitis, retinitis or fatal haemorrhagic fever may occur [6]. Due to direct transmission from ruminant to humans, human infection is linked to occupational qualifications (veterinarian, butcher, breeders) [7]. Despite a wide number of mosquito species involved in the transmission of the RVF virus (RVFV), and because of the vertical transmission of RVFV demonstrated in *Ae. mcintoshi* in Kenya, mosquitoes of the *Aedes* genus are considered to be responsible for the initiation of the RVF outbreaks and the persistence of the virus in the field during inter-epizootic periods in eastern Africa [8]. Vertical transmission has never been demonstrated in *Culex* mosquitoes, which are responsible for virus amplification during outbreaks [6]. Wild terrestrial mammals are suspected to play a role in the maintenance of the RVFV during inter-epizootic periods, but no reservoir species has been identified to date [9].

First described in 1930 [10] RVF infection has been recorded in most of the African countries from the Cape of Good Hope to the Nile delta [11]. RVF had been reported for the first time outside the African continent in 2000 when it spread to the Arabian peninsula [12]. During the last decade, outbreak frequency has increased in Africa and the Indian Ocean: in 2006–2007 in Eastern Africa (Kenya, Somalia and Tanzania) [13], [14], in 2007 in Sudan [15], [16] and Mayotte [17]. In Madagascar, RVFV was first isolated in 1979, from mosquitoes trapped in the Perinet forest (Moramanga District) without any reported human nor animal clinical case [18], [19]. The first outbreak occurred during the rainy season of 1990–1991 [20] in both human and animal populations. The last outbreak occurred in 2008–2009, during two consecutive rainy seasons, in the whole country [21]. Numerous cases in human (418 reported cases of which 59 were laboratory-confirmed) and in ruminants were reported. During this outbreak, Madagascar highlands were heavily affected [22]. A three-year serological follow-up in cattle (2009–2011) conducted in a pilot area of Madagascar highlands suggested a recurrent transmission of the virus in this temperate area [4], [22], [23]. An entomological study performed in the same area showed that *Aedes* mosquitoes were too rare to explain the recurrent virus circulation. Furthermore, because of the unfavourable meteorological conditions linked to elevation, mosquitoes were absent or rare during the cold and dry season and had a relatively low population density during the warm and wet season [24], [25]. Besides, a serological and virological survey in 963 small terrestrial mammals of the area belonging to 18 species (11 *tenrecidae*: 11 species, *nesomyidae*: 6 species, *muridae*: 1 species, pers. communication: Olive M.M.), showed that RVF did not circulate in these animals [26].

The objectives of this study were to propose a model of RVFV transmission, and to use this model to infer on mechanisms involved in the recurrent transmission of RVFV in this temperate and mountainous area. To achieve these goals a spatialized metapopulation model was designed and parameterized using data collected during field studies conducted between 2009 and 2011 [4], [22]–[24]. This deterministic model incorporated cattle populations living in villages and vector populations breeding in rice fields. Cattle populations were connected by the cattle trade and barter network driving cattle movements between villages, and exposed to vector populations living in rice fields surrounding the corresponding villages [23]. The model was used to analyse three potential scenarios that could explain the recurrent circulation of RVFV in the study area: (i) RVFV overwintering thanks to a direct transmission between cattle, vectors being absent during winter, (ii) a low level vector-based circulation during winter thanks to a residual vector population, without direct transmission between cattle, (iii) a combination of both above mentioned mechanisms.

## Materials and Methods

### Study area

**Figure 1:**
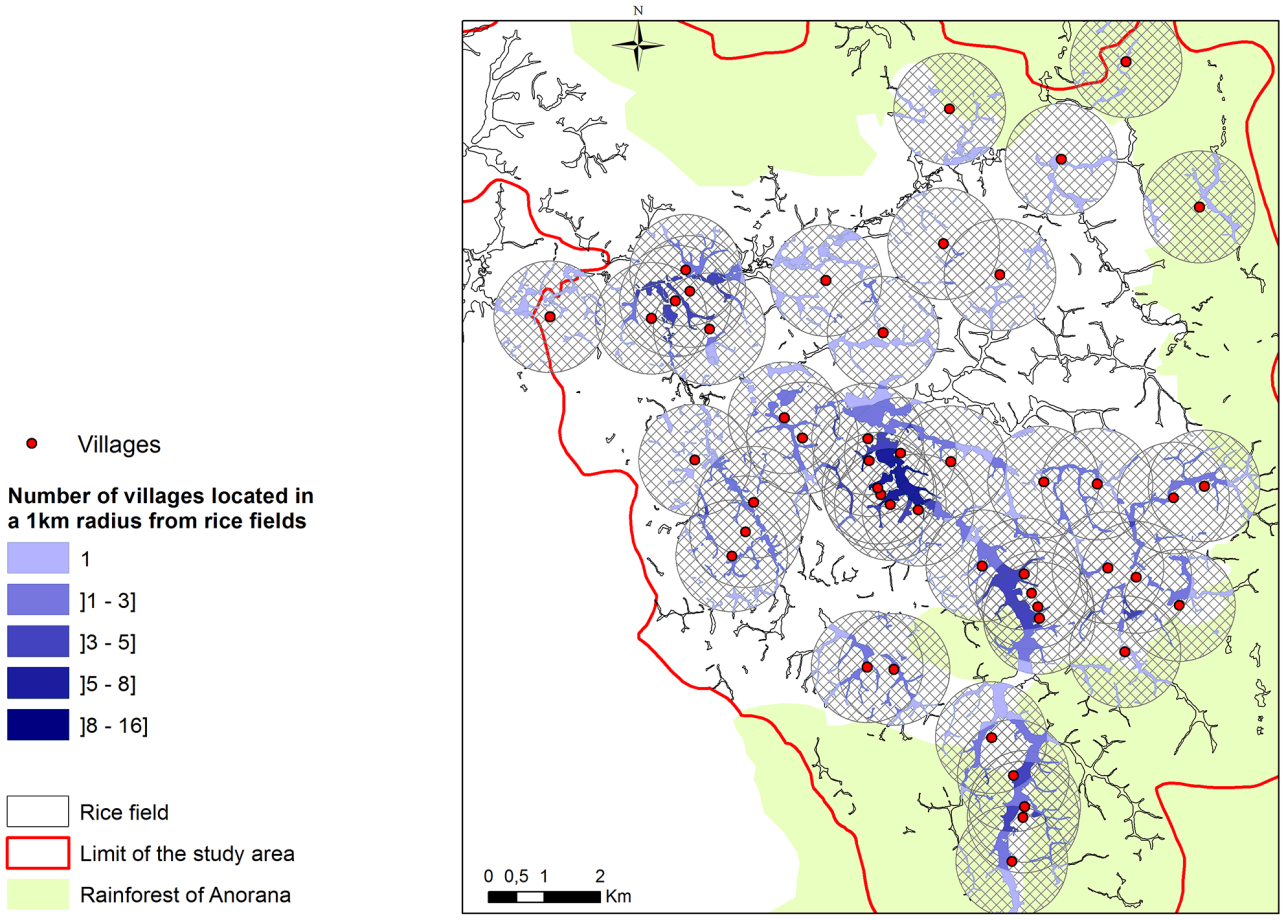
Location of the villages and rice fields in the study area. The color gradient of the rice field indicates the number of villages at less than 1km. Circular areas around villages have a 1 km radius.

The study area was an area of 200 km^2^ (GPS coordinates: 47,967; −18,337) covered by crops and rice fields and surrounded by the rainforest corridor of Anorana (Fig. 1). This area is located about 1200m above the sea level [22], [23] and connected with the largest rice growing area of Madagascar. The human population size was estimated at 6500 persons in 2011. The same year, the small ruminant (sheep and goat) and the cattle populations were estimated to be 60 and 2140 heads respectively (census performed by veterinary services). This area is characterized by two distinct climatic seasons: the rainy season starts in October and ends in April, and the dry season runs from May to September, the lowest winter temperatures being concentrated in July and August.

Rice production induces an agricultural and commercial seasonality in the study area. The main period for transplanting rice starts in October and continues until the end of January, overlapping with the rainy season. Some agro-breeders may transplant an additional time from July to September when rice fields are located near permanent water. Harvest is performed from the end of February until May. Some breeders, whose fields are close enough to a permanent water point, can successfully transplant rice from July to August, and make a second harvest from December to January. Transplanting and harvest periods are periods of intense work that induce seasonal labor needs and cattle for pulling force. To get cattle adapted to rice work, breeders have to replace their animals when they become too old. This renewal may be performed thanks to the usual cattle trade that consist in buying or selling a cattle, when breeders have available money, i.e. after having sold their rice, during the dry season. Besides usual trade, cattle renewal may also be performed thanks to a specific, traditional, exchange practice consisting in an animal barter without any money transaction. This barter induces symmetrical movements of cattle: a breeder who barters an animal always receives another animal in return. However, to conclude the barter, the applicant has to exhibit his cattle to other breeders after the working day, near rice fields. This leads to an increased exposure of applicant's herd to mosquito bites and to animals of other breeders [23].

### Data

Due to the very small number of goats and sheep in the area we focused this study on the cattle population.

A first serological survey was conducted in 2009 in 984 animals of 43 villages of the study area. A seroprevalence rate of 28% (95% CI: 25–31%) was observed, that varied between 0% and 71.4% according to the village [22]. A census of the cattle population was performed in each village: the number of cattle and cows belonging to each breeder was recorded. In 2010, a second serosurvey conducted in 484 animals of 39 villages showed a seroconversion rate of 7% (95% CI: 5–10%) [23]. This seroconversion rate ranged from 0% to 20% according to the village [23]. Movements of cattle in the study area were also documented: renewal practices of each breeder (usual trade, barter) and number of animals sold, bought or bartered in the preceding year, as well as the origin of the introduced animals. This allowed generating the matrix of animal movements between villages, containing, for each pair of villages (A, B), the number of animals moved in one year from village A to village B. The study also identified the villages where animals bought on markets (located outside the study area) had been introduced and the number of animals introduced in one year from these markets. During the cattle exchanges survey, 48 villages were investigated among the 52 of the area.

In 2011, a monthly longitudinal follow-up in 4 villages of the area was performed. Cattle population of each village was monitored: birth, death, disease and aborting events were registered monthly. Among seronegative cattle ears-tagged in May 2010 [23] and that could be tested in January 2011, the observed seroconversion rate was 14% (n = 59). During the follow-up, approximately 100 blood samples were taken each month, and seroconversions were observed in 4 animals, viral RNA being detected in 2 of them [4].

During the 2010 serosurvey and the 2011 longitudinal follow-up, a small number of abortion cases in pregnant animals and of mortality in young animals were reported by the breeders, often due to accidental causes. None of these cases could be attributed to RVF. In particular, for abortion cases, available serological results did not show any seroconversion.

An entomological study was conducted in 2009–2010 in the same area to investigate species diversity and abundance of potential RVFV vectors in different biotopes [24]. Captures were carried out on a monthly basis: more than 56,000 adult mosquitoes of 35 different species were collected. *Aedes* mosquitoes were rare. The most abundant captured mosquitoes were *Culex pipiens, Culex antennatus, Culex univittatus, Anopheles squamosus* and *Anopheles coustani*. Rice field are the larval habitat of the above mentioned mosquito species and their population dynamics in the area were similar [24]. We thus considered a single biotype for vectors. We also considered rice fields as a homogeneous biotope that represents the potential sources of exposure to mosquito bites. Using aerial pictures (300*300 ppp) obtained from the National land plan (PNF) of Madagascar (June 2011), rice fields of the study area were digitized using ArcGIS 10 software. The villages of the area were geolocated using Global Positionning System (GPS).

### Ethics statement

For cultural reasons, no ethical body was involved in the study protocol. However, data collections, blood samples and protocol were done in collaboration with the Malagasy Veterinary Services and according to Malagasy regulations about animal welfare. Serological products were treated according to international regulations. Meetings were organized with breeders of the study area to explain the goals of the study and the decision to participate was taken individually according to the breeders' willingness and at the village level (all villages were visited). Breeders gave the permission to use their animals for the whole study and could stop at any time. Informed consent was given orally and documented in questionnaires. Written consent could not be obtained for the same cultural reason.

### Model description

**Figure 2:**
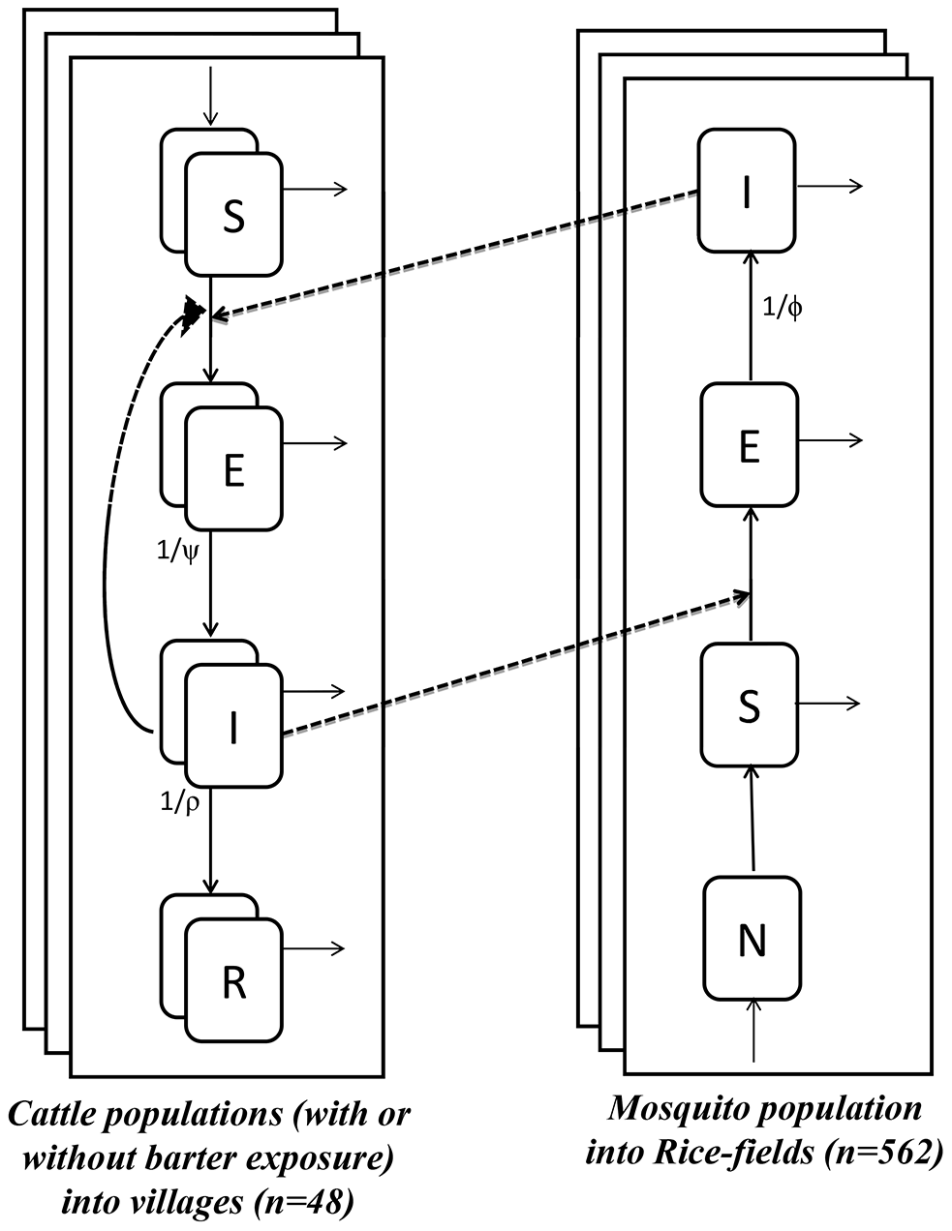
Graphical representation of the model. Cattle population is divided into susceptible (*S*), incubating (*E*), viraemic (*I*) and immune (*R*) individuals in each village. Two populations per village were considered depending on whether or not animals are exposed to the barter practice. Mosquito population is divided into nulliparous (*N*), parous and non-infected (*S*), parous and infected, but non-infectious (*E*, during the extrinsic incubation period), and parous, infected and infectious (*I*, after the end of the extrinsic incubation period) in each rice field. Full dark arrows represent transition from on state to the other. Full thin arrows represent demographic processus of birth and death specific to both metapopulation. Dotted lines represent infection dynamics. The full description of the parameters can be found in S1 Text. 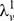: force of infection due to direct transmission into village v, 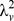: force of infection due to vector based transmission into village v, 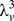: average force of infection due to vector based transmission into villages accepting the barter into village v (See S1 Text for development of force infection expressions related to cattle exchange practices), 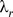: force of infection for mosquitoes of rice field r.

The model was a deterministic model operating in discrete time with daily time steps (Fig. 2, see S1 Text for model equations). The modeled epidemiological system was a metapopulation composed of cattle populations living in villages, exposed to bites of mosquito populations living in neighboring rice-fields. Both population types were spatially explicit. In the absence of serological evidence of RVFV in wild small mammals of the area [26], no wild reservoir was considered. Similarly, because mortality in young animals was very low during the study period, and as none of the few abortion cases was attributed to RVF, we didn′t consider disease-induced abortion or mortality in the model. A concise description of the model is given below, the full formal description being given in the supplementary materials (S1 Text).

In each village, the cattle population was structured according to the breeder renewal practices (trade alone or associated with barter) and to the individual health state: susceptible (*S*), incubating (*E*), viraemic (*I*) and immune (*R*). Transitions from *E* to *I* and from *I* to *R* were assumed to occur at constant rates (denoted 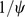 and 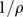, where 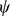 and 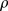 were the duration of the incubation period and of the viraemia, respectively). Two RVFV transmission modes were considered: vector-based transmission and direct transmission. Direct transmission was assumed to occur when viraemic cows calve, according to a transmission parameter denoted 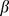. The exposure of cattle to mosquito bites was assumed to vary according to the breeder renewal practices. For cattle belonging to breeders who did not perform barter, 100% of the exposure to mosquito bites was attributed to mosquito populations of rice fields surrounding the village. Conversely, for cattle belonging to breeders who barter animals, only part of this exposure was attributed to local vector populations. The remainder part (denoted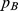) was attributed to mosquito populations surrounding the distant villages where the breeder used to go when he tried to barter some of his animals. The force of infection induced by vector-based transmission was the product of the vector/host ratio, considering the mosquito production of 1 m^2^ of rice field (denoted 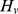), by the area of the above rice fields and the daily relative abundance of infectious mosquitoes in these rice fields. The mortality induced by RVFV was neglected. Demographic processes were taken into account, with a per-capita birth rate denoted 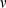 and a mortality rate (for other causes than RVF) denoted 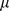. Animal movements (either bartered or sold) were assumed instantaneous.

In each rice field, the mosquito population was structured according to the health state: nulliparous (*N*), parous and non-infected (*S*), parous and infected, but non-infectious (*E*, during the extrinsic incubation period), and parous, infected and infectious (*I*, after the end of the extrinsic incubation period). The transition from *E* to *I* was assumed to occur at a constant rate (denoted 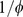, where 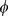 was the duration of the extrinsic incubation period). The transition rate from *N* and *S* to *E* was the product of the proportion of mosquitoes that take a blood meal (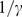, where 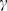 was the duration of the gonotrophic cycle) by the proportion of viraemic cattle among those that may be bitten by the mosquitoes of the rice field, either because they live in the neighboring villages, or because they have been brought there by a breeder that wants to barter some of his animals. Because of the rarity of *Aedes sp*, vertical transmission was not considered. The mosquito population dynamic (oviposition, larval development, emergence) was forced by two time-varying parameters: the daily relative abundance of mosquitoes (denoted 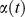), and the parous rate (the proportion of female mosquitoes that have laid eggs at least once, denoted 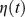).

### Parameterization

#### Structure of metapopulation and initial conditions

The mosquito species observed in the study area have a flight distance of about 1 km. The vector populations responsible for vector-based transmission in a given village were thus assumed to be those of rice fields located within a radius of 1 km around that village. Because rice fields could be in a 1 km radius of several villages, the corresponding vector populations could support RVFV circulation between villages. A buffer zone of 1 km radius was thus created around each village, and the spatial intersection between these buffers was computed. The resulting buffer fragments were then used to compute their intersection with rice culture areas. This resulted in 562 rice fields (or fragments of rice fields) located <1 km far from one or several villages (Fig. 1). A unique ID was assigned to each. For each village *x*, the set of rice fields located in a 1 km radius was computed. Similarly, for each rice field *y*, the set of villages located in a 1 km radius was computed.

We assumed that the study area was initially free of RVFV. The date of virus introduction was denoted *T_0_*. It was assumed that a unique incubating cattle (in the *E* state) was introduced, this initial amount of infectivity being shared between the villages in which some breeders used to buy animals from distant markets, according to the data collected in 2009. The duration of RVF incubation is short and field data showed that the yearly number of cattle introduced into the study area from distant markets was low. Therefore, the introduction of RVFV was assumed to be a unique event, occurring only once.

#### Fixed parameters

Observational data collected in 2009 were used to set the number of cattle and cows per village and per group of breeders (defined based on renewal practices), and the average number of cattle moved from one village to another, either by usual trade or by barter. The monthly follow-up performed in 2011 allowed estimating daily per-capita calving (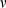) and death rates (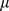). Neither calving nor mortality was seasonal in the study area. The duration of incubation (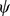) and viraemic (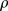) periods were set according to literature (Table 1).

**Table 1:** Parameters of the deterministic metapopulation model.

Parameters	Notation	Value	Unit (range)	reference
**Cattle population**				
Birth rate (annual)	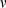	0.33	rate (0–1)	field data
Mortality rate (annual)	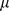	0.06	rate (0–1)	field data
Incubation period	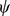	4	days	[5]
Viremia	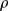	4	days	[11]
Proportion of exposure to mosquito bites attributed to distant rice fields for cattle that belong to breeders who use to barter animals ( 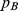 )	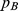	10%	percentage	field observations
**Vector population**				
Duration of gonotrophic cycle	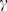	3	days	[24], field data
Duration of extrinsic incubation period	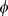	7	days	(*Fontenille* pers. com)
Parous rate	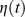	0.56+0.02j (with j = 0 for Oct. 1)	rate (0–0.9)	[24], linear regression from field data
Maximal limit of the parousrate	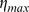	0.9	rate	[24], field data
Duration of the warm and wet season	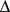	October 1 to April 30	day	[24], field observations
Relative abundance of mosquitoes during the warm and wet season	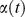	Trapezoidal form with maximum ( 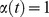 ) from January 1 to March 31	Proportion	[24], field data

The mosquito species observed in the area are active at dusk and during the night. Field observations performed during the 2011 follow-up showed that breeders wanting to barter some of their animals did it after the working day, at dusk. Afterwards, they came back to their village. Therefore, the exposure of their cattle to mosquito bites mainly occurred in their village, and the proportion occurring near the villages with which the breeder used to barter animals (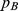) was set to 10%.

The daily variations of the relative abundance of vectors (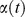) were set to reproduce a typical yearly dynamic, linked to seasonality. During the cold and dry season (May 1 to September 30), abundance was assumed to have a low (possibly null) level, denoted 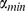. Then it was assumed to increase linearly until December 31, reaching a plateau of maximal relative abundance (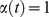) in January, February and March. The relative abundance then decreased linearly in April to reach again the lowest level 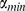 at May, 1.

The daily variations of the parous rate (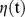) were assumed to show a linear increase during the wet and hot season (October 1 to April 30). The initial value of the parous rate (0.56 for October, 1) and the slope of the linear increase (0.002) were computed from field data collected during the 2009–2010 entomological survey. The parous rate was assumed to show a plateau once having reached a maximal value, denoted 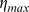. This maximal value was set to 0.90, the maximal value observed during the entomological survey. The duration of the gonotrophic cycle (*γ*) and that of the extrinsic incubation period (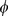) were set according to literature to 3 and 7 days, respectively (Table 1).

#### Estimated parameters

Three parameters were estimated using seroprevalence data (n = 81 village-level seroprevalence data: the date, the number of positive and of tested animals) and seroconversion data (n = 109 village-level seroconversion data: two dates, the number of animals initially seronegative and, among them, the number of observed seroconversions) collected in the study area between 2009 and 2011: the vector-host ratio (*H_v_*), the relative abundance of vectors during the cold and dry season (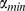), and the direct transmission parameter (*β*). For a given value of the triple (*H_v_*, 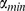, *β*), the model was used to compute predicted seroprevalence and seroconversion rates homologous to the observed seroprevalence and seroconversion data (i.e. for the same villages and the same dates and periods). The likelihood of observed data was the joint binomial probability to observe the seroprevalence and seroconversion data, conditionally to these predicted seroprevalence and seroconversion rates. The log-likelihood was then minimized to obtain estimates of *H_v_*, 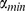, and *β*. The variance-covariance matrix was obtained by inverting the Hessian matrix. It was used to compute the confidence intervals or parameter estimates, and to plot the 3-dimensional confidence region of these estimates.

### Model exploitation

To investigate mechanisms that could explain the observed recurrent circulation of RVFV in the study area between 2008 and 2011, 4 models were implemented.

- M0: the null model: no active residual vector population in winter (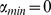) and no direct transmission (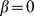); a single parameter was thus estimated: *H_v_*,
- M1: overwintering thanks to direct transmission (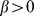) but without active residual vector population in winter (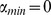); two parameters were estimated: *H_v_* and 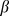,
- M2: overwintering thanks to an active residual vector population in winter (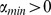) but without direct transmission (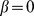); two parameters were estimated: *H_v_* and 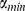,
- M3: both mechanisms (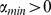
and 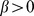); three parameters were estimated: *H_v_*, 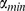 and 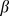.

In the study area, the first clinical cases in cattle and human were reported and confirmed by the Pasteur Institute of Madagascar in February 2008 [21]. Field data showed that cattle purchase from distant markets mainly occurred after the rice harvest (when breeders have money available), during the dry and cold season (May to September). Eight dates were thus considered for virus introduction (*T_0_*): the first day of each month between June 2007 and January 2008. Because RVFV circulation cannot start during the dry and cold season for M0, only 4 dates were considered for that model: 2007-10-01, 2007-11-01, 2007-12-01 and 2008-01-01. Parameter estimation was thus performed for 28 models (M0 for 4 introduction dates; M1, M2 and M3 for 8 introduction dates). These 28 models were ranked according to the Akaike Information Criterion, corrected for small samples (because the number of parameters *k* was small compared to size of the dataset *n*: 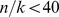): 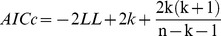 where *LL* is the minimal log-likelihood, *n* is the number of observations (81 seroprevalence data and 109 seroconversion data), and *k* is the number of parameters of the model (1 for M0, 2 for M1 and M2 and 3 for M3). Multimodel inference methods were then used to combine the 28 models [27], [28]. A weight was first computed for each of them: 
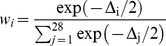
, with 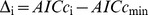, where 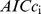 is the *AICc* of the i^th^ model, and 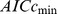 is the minimal value of *AICc* among the 28 models. By construction, these weights verify: 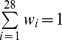. The smallest subset *R* of models verifying 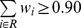 was then selected, that represented a “confidence set” of models. The weights of the corresponding models were used to compute weighted values of model coefficients and the associated variances. Similarly, weighted predictions were computed to analyze the quality of model fit, to describe the overall dynamic or RVFV circulation in cattle and vectors, and to compute the proportion of cattle infections due to direct transmission *vs* vector-based transmission.

The core of the model was programmed using the Java language. It was embedded in the R environment with which statistical analyses were performed [29]. The source code is available upon request.

### Sensitivity analysis

A systematic sensitivity analysis was conducted to study the independent effects of 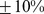 parameter variations of fixed parameter values: cattle birth (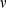) and mortality rates (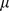), duration of incubation (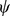) and of viraemia (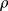) periods, proportion of exposure to mosquito bites attributed to distant rice fields for cattle that belong to breeders who use to barter animals (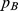), duration of the extrinsic incubation period (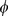) and of the gonotrophic cycle (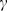), maximal value of the parous rate (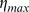), and duration of the wet season (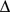) (Table 1). For each of the 18 corresponding parameter sets (9 parameters and 2 values per parameter), parameter estimation was performed as described above for the 28 studied models, and the above multimodel inference methods were used to compute the composition of the “confidence set” of models, as well as the overall proportion of cattle infections due to direct transmission *vs* vector-based transmission.

## Results

Of the 28 models compared, the model with the lowest AICc value was M3 (direct transmission when viraemic cows calve combined with a persistence of mosquito-based transmission in winter), with a RVFV introduction on 2007-09-01 (i.e. at the end of the dry and cold season and the period of rice transplant), approximately 6 months before the first laboratory-confirmed clinical cases were reported (February 2008) (Table 2). The 90% confidence set of models included the above model (weight: 0.73) and a second one: M3 with a RVFV introduction on 2007-10-01 (weight: 0.24). These two models achieved collectively a cumulated weight of 0.97 (the 26 remaining models weighing thus collectively only 0.03).

**Table 2:** Comparison of the values of the Akaike information criterion (corrected for small sample sizes), for the models M0 (no direct transmission, no residual mosquito population in winter), M1 (direct transmission when viraemic cows calve, no residual mosquito population in winter), M2 (no direct transmission, residual mosquito population in winter) and M3 (direct transmission when viraemic cows calve and residual mosquito population in winter) and for eight possible dates for the introduction of the RVFV in the study area.

RVFV introduction date	M0	M1	M2	M3
2007-06-01		789	835	752
2007-07-01		801	832	748
2007-08-01		825	827	742
2007-09-01		866	818	735
2007-10-01	902	810	831	737
2007-11-01	844	777	934	754
2007-12-01	857	789	971	756
2008-01-01	880	813	884	758

**Figure 3:**
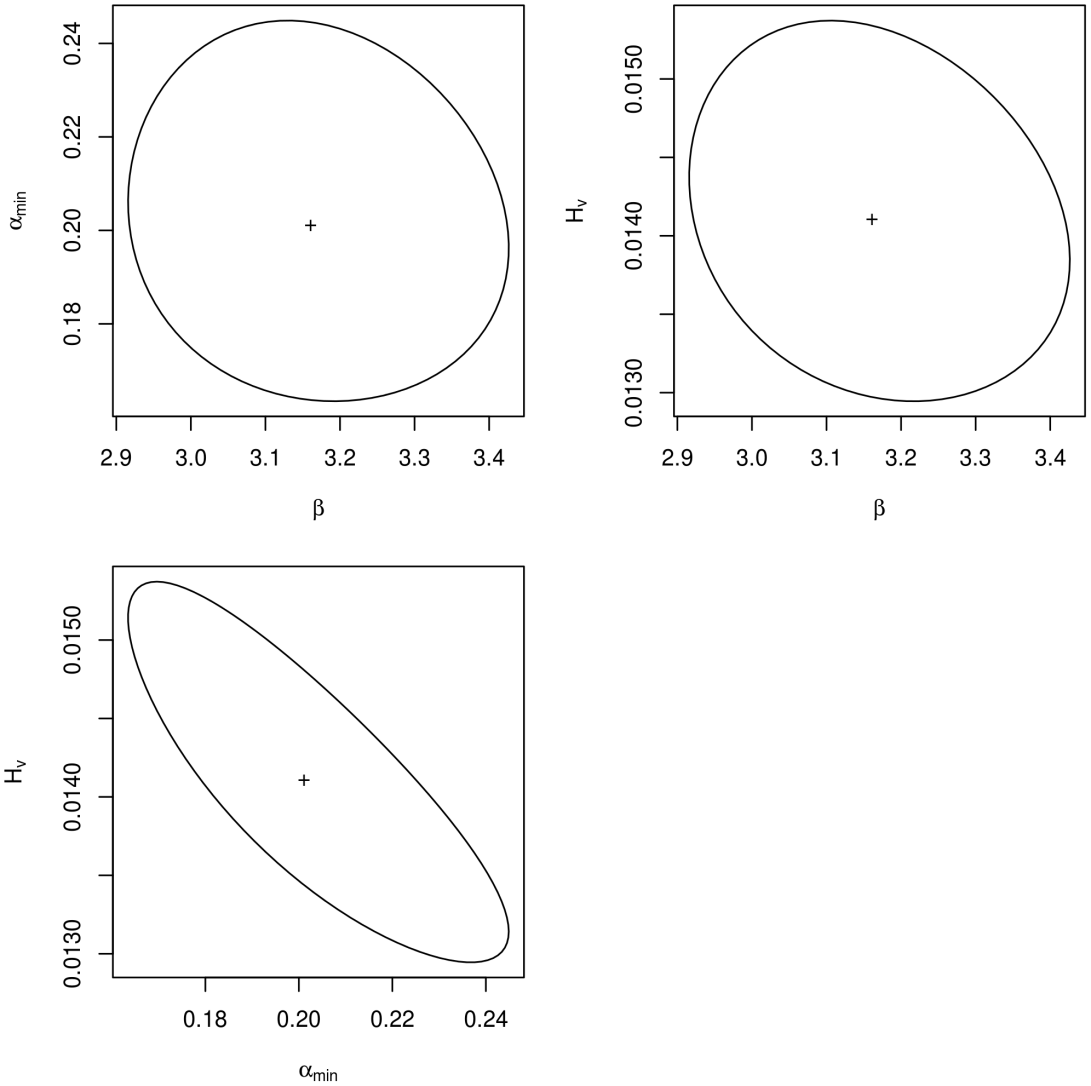
Confidence regions of the weighted parameter values (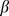: direct transmission when viraemic cows calve, 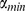: relative abundance of vectors during the dry and cold season, *H_v_*: vector/host ratio).

**Figure 4:**
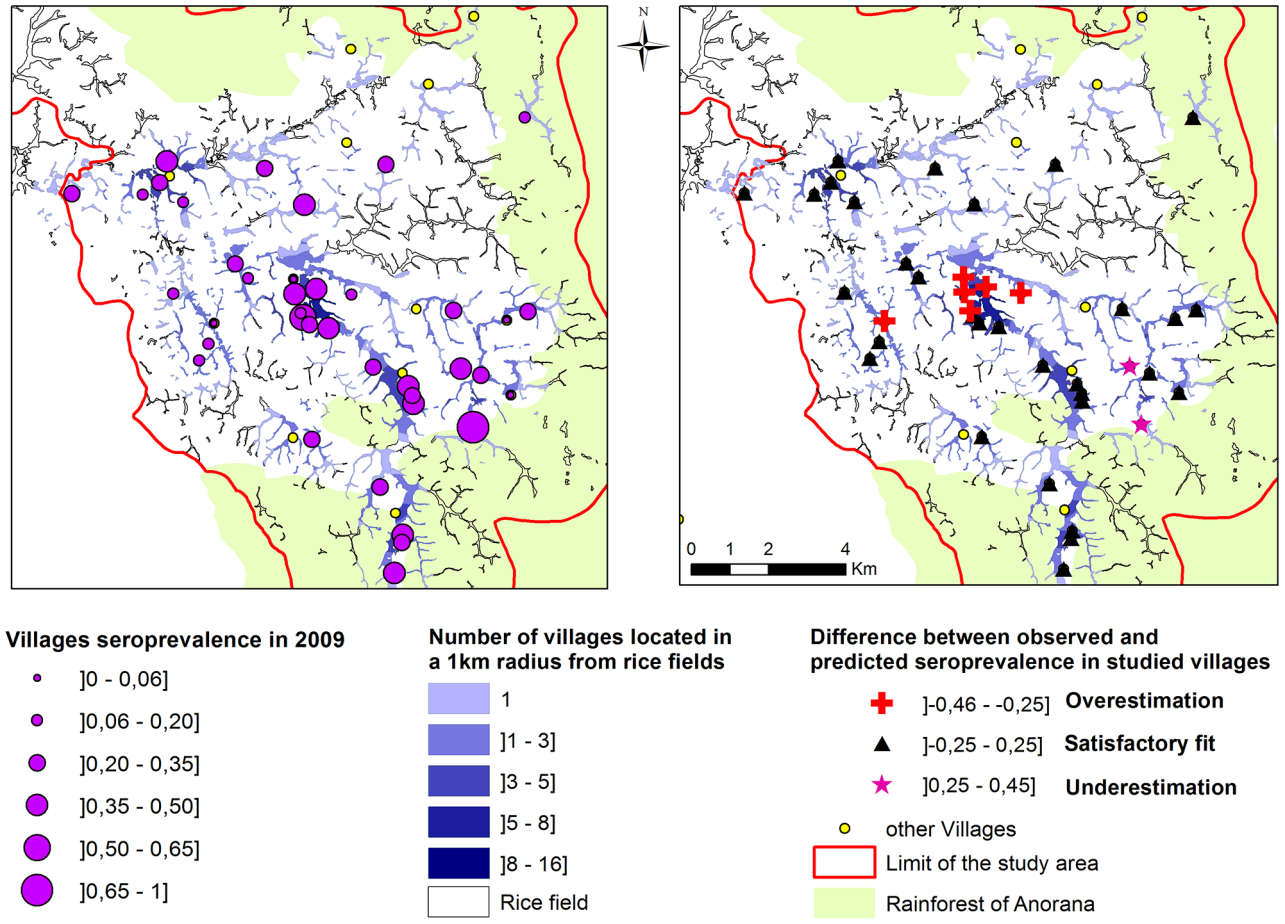
Observed seroprevalence in 2009 (left) and difference between observed and predicted seroprevalence in 2009, in the studied villages. Villages with predicted seroprevalence higher than observed seroprevalence (red cross) were aggregated in the middle of the area where most rice fields are close to several villages. Villages with predicted seroprevalence lower than observed seroprevalence (pink star) were less aggregated. For most villages the differences between predicted and observed seroprevalence were low (triangle).

The weighted parameter values (Table 3) accounted for a significant direct transmission when viraemic cows calve (with approximately 3 animals exposed to RVFV at each viraemic cow calving), and a persistence of the mosquito population during the dry and cold season at a significant level (20% of the maximal abundance in the January-March period), that maintained the vector-borne transmission. The shape of the confidence region of the weighted parameters (Fig. 3) indicated a low covariance between the direct transmission parameter (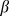) and the vector/host ratio (*H_v_*), as well as between *β* and the relative abundance of mosquito during the dry and cold season (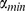). Conversely, 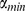 and the vector/host ratio appeared linked, with lower values of *H_v_* when the value of 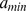 increased. Weighted predictions were computed for the village-specific seroprevalence and seroconversion rates. Based on these rates and on the number of tested animals per village, we computed the binomial confidence intervals of the predicted number of positive animals in each village (either seropositive animals, or animals with seroconversion). A good fit of the model was thus observed at the village level (Fig. 4). For 79% of the 81 villages in which seroprevalence data were available, the observed number of seropositive animals was included in the confidence interval of the predicted value. Furthermore, for 89% of the 109 villages in which seroconversion data were available, the observed number of animals having seroconverted was included in the confidence interval of the predicted value. An aggregation of villages where the predicted seroprevalence rate was higher than the observed value was however observed in the centre of the studied area, around the rice fields that were close to several villages (Fig. 4).

**Table 3:** Weighted values of model parameters for model M3 (direct transmission when viraemic cows calve and residual mosquito population in winter) and an introduction of RVFV on 2007-09-01 (weight: 0.73) or on 2007-10-01 (weight: 0.24).

Parameter	Notation	Estimated value	95% confidence interval
Direct transmission when viraemic cows calve	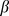	3.16	[2.96–3.37]
Relative abundance of vectors during the dry and cold season	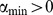	0.20	[0.17–0.24]
Vector/host ratio (vector production of 1 m^2^ of rice field)	*H_v_*	0.014	[0.013–0.015]

**Figure 5:**
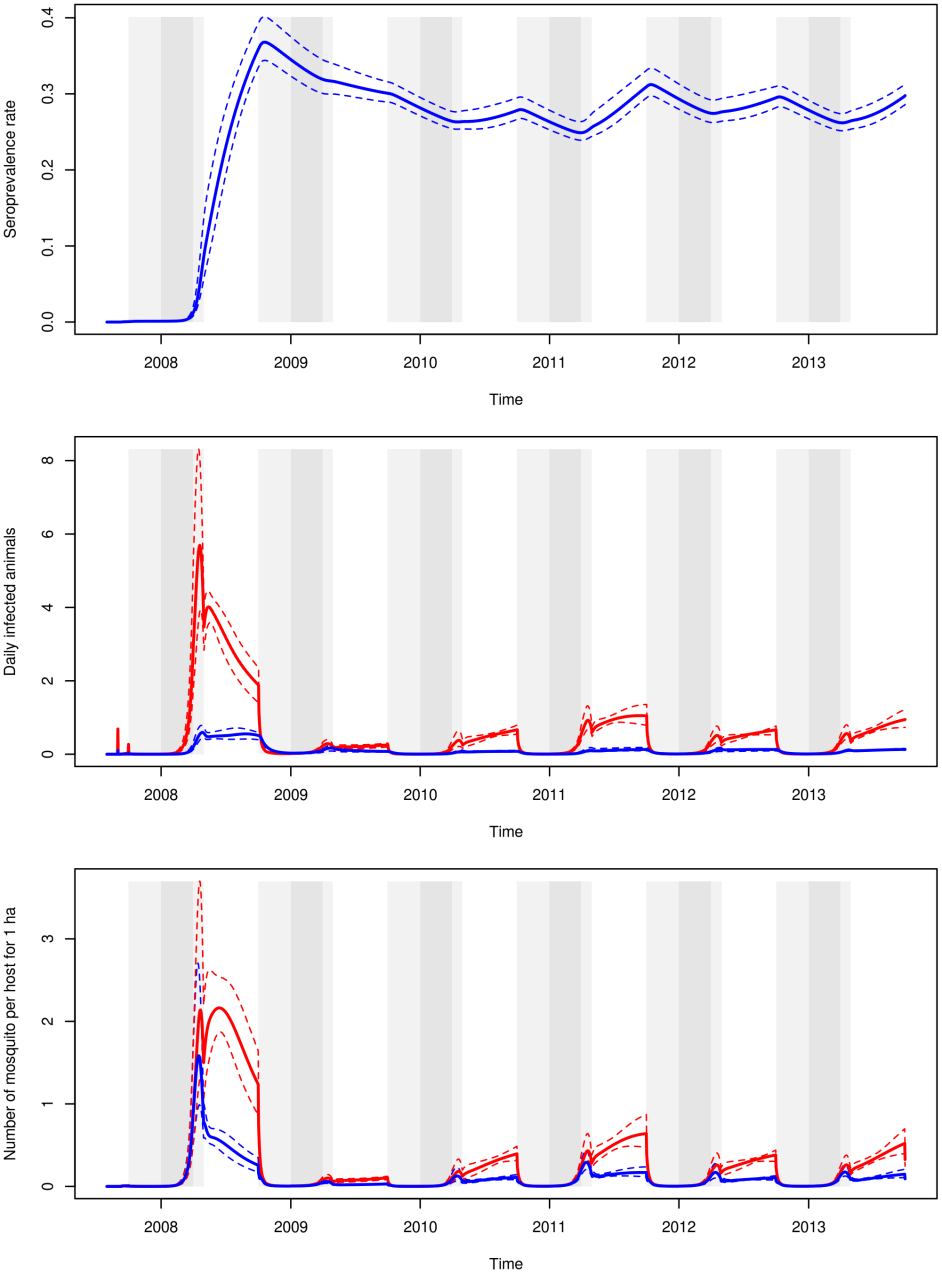
Weighted predictions of the evolution of the global seroprevalence rate (top), of the daily number of infected cattle due to direct (blue) and vector-borne (red) transmission (middle), and of the number of mosquitoes per host that are in the extrinsic incubation period (E state, blue) and infectious states (I state, red) (bottom). Dashed lines: confidence area of the weighted predictions. Grey areas: the rainy seasons (peak: dark grey and beginning/end: light grey).

The weighted predicted dynamics of RVFV circulation in the cattle and mosquito populations of the study area is shown in Fig. 5. According to the predicted dynamic, RVFV kept circulating after the 2008 epidemic peak but at a low level, vector-borne transmission being the main RVFV transmission route. The number of cattle infected by vector-borne transmission was predicted to be similar during the wet and warm season and during the dry and cold season (Table 4). Due to the high parous rate of the residual mosquito population, the number of cattle infected during the dry and cold season remained high despite the mosquito abundance during that season was 20% of the abundance during the wet and warm season. The number of cattle infected by direct transmission (when viraemic cows calve) was globally similar for both seasons (Table 4). Sixteen per cent of the infected cattle were predicted to have been infected by direct transmission, against 84% by vector-borne transmission, for the whole period (Table 4). However, direct transmission was predicted to play a significant role immediately after the epidemic peak with>40% of infections occurring by direct transmission during the next wet and warm season.

**Table 4:** Weighted prediction of the number of animals infected by direct transmission, when viraemic cows calve, and by vector-borne transmission, according to the season (dry and cold season, May-September, wet and warm season: October-April).

Season	Year	Infections caused by vector-borne transmission	Infections caused by direct transmission	Proportion of infections caused by direct transmission (95% CI)
dry and cold	2007	0.35	0.11	0.23 [0.21–0.26]
wet and warm	2007–2008	294	32	0.10 [0.08–0.11]
dry and cold	2008	332	64	0.16 [0.13–0.20]
wet and warm	2008–2009	32	27	0.46 [0.41–0.47]
dry and cold	2009	27	11	0.29 [0.25–0.33]
wet and warm	2009–2010	25	6	0.20 [0.16–0.24]
dry and cold	2010	65	9	0.12 [0.11–0.15]
wet and warm	2010–2011	61	8	0.12 [0.09–0.18]
dry and cold	2011	121	14	0.11 [0.08–0.15]
wet and warm	2011–2012	40	12	0.24 [0.17–0.28]
dry and cold	2012	69	16	0.18 [0.14–0.20]
wet and warm	2012–2013	40	11	0.21 [0.17–0.23]
dry and cold	Whole period	614	114	0.16 [0.13–0.19]
wet and warm	Whole period	492	96	0.16 [0.14–0.19]
Total	Whole period	1106	210	0.16 [0.13–0.18]

The sensitivity analysis confirmed the above results. Whatever the tested parameter set (n = 18, i.e. 9 parameters and 2 values per parameter), only M3 (direct transmission when viraemic cows calve combined with a persistence of mosquito-based transmission in winter) was included in the confidence set of models, that contained 2 or 3 models. Introduction dates included 2007-09-01 and 2007-10-01 for 16 of the 18 parameter sets (i.e. the same dates as those obtained for the default parameter values). The two remaining parameter sets corresponded to changes of the maximal value of the parous rate (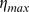), for which the confidence set of models combined M3 with several other RVFV introduction dates: 2007-06-01, 2007-07-01, 2007-11-01 and 2007-12-01). Finally, 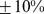 variations of parameter values had a low impact on the overall proportion of cattle infections due to direct transmission. Whatever the modified parameter, the absolute difference of this proportion was always <1%, except when the maximal value of the parous rate (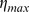) was decreased, the proportion of cattle infections due to direct transmission being increased by 10% (i.e. 26% instead of 16% with the default parameter values).

## Discussion

In the temperate and mountainous ecosystem of Madagascar highlands, the high altitude and low temperature, especially during the dry and cold season, make climatic conditions unfavourable to a yearlong mosquito proliferation, and thus to a permanent RVFV circulation. Nevertheless, successive serological surveys conducted in a pilot area after the 2008 epidemic peak showed a recurrent circulation of RVFV. A model was elaborated to mimic RVFV transmission between cattle and mosquito populations in villages and rice fields of the study area. Data collected during field investigations were used to parameterize this model. The model was then used to investigate possible mechanisms explaining the recurrent circulation of RVFV in the study area. Two mechanisms were considered: RVFV circulation during the dry and cold season thanks to an active residual mosquito population, and direct transmission of RVFV between cattle when viraemic cows calve. Twenty-eight models were compared that combined these mechanisms with 8 possible RVFV introduction dates. Multi-model inference techniques (based on the Akaike information criterion) allowed to select a subset of two optimal models among these 28 models. Both included a significant active residual mosquito population during the dry and cold season (20% of the maximal mosquito abundance, during the wet and warm season), and a significant level of direct transmission of RVFV between cattle when viraemic cows calve (with approximately 3 animals exposed to RVFV infection for each calving of a viraemic cow). Model fit was judged satisfactory as, for>75% of seroprevalence and seroconversion data, the fitted rate was within the confidence interval of the observed rate. According to the predicted dynamic, vector-based transmission remained the major RVFV transmission mode in the area (responsible for 84% of infections in cattle), even if the direct transmission played a significant role (16% of infections in cattle), especially during the second year.

Even if it has never been demonstrated, direct transmission between cattle seem plausible. Indeed, RVFV infection in human is often attributed to contacts with abortion products or with cattle body fluids. Due to the behaviour of cattle and to the close contacts between cows in night pens of the study area villages, direct transmission at calving of viraemic cows appears probable. Considering the important consequences this transmission route would imply on RVF circulation and spread, further field and experimental studies are needed to confirm or invalidate it.

Other transmission modes between cattle could also play a role in RVFV circulation. Vertical transmission of RVFV was experimentally demonstrated in ewes, in the absence of clinical signs or detectable maternal viremia [30]. To explain the recurrent circulation of RVFV, the existence of a wild reservoir has been suspected. However, field studies conducted in the area did not allow identifying any serologically or virologically positive wild small terrestrials mammal [26]. Besides the existence of an active residual mosquito population during the dry and cold season, other mechanisms based on vector biology could explain the recurrent circulation of RVFV in the study area. Vertical transmission has been demonstrated in mosquito of *Aedes* genus; however entomological field studies conducted in the area did not allow trapping mosquitoes of this genus. Observed mosquitoes rather belonged to *Culex* and *Anopheles* species, in which RVFV vertical transmission has never been demonstrated. Overwintering in adult *Culex* females (as it is the case for West Nile virus (WNV) [31]) has never been demonstrated for RVFV, but certainly deserves to be studied in the vector species of this altitude area. Non-mosquito vectors could also play a role in the RVFV cycle of Madagascar highlands, such as ticks [32] or *Stomoxys* flies [33]. According to Baldacchino and colleagues [33], *Stomoxys calcitrans* should be considered a possible mechanical vector of RVFV because of its close association with domestic animals that serve as amplifying hosts. For the same reason, ticks could play a role in RVFV transmission and persistence [34]. Transstadial and horizontal transmission was experimentally demonstrated in *Hyalomma truncatum* [32], and the geographical distribution of this species has been associated with the incidence of RVF in Africa [34]. However, despite the frequent detection and introduction from the African continent during the last 50 years, *H. truncatum* did not colonize any region of Madagascar. In the study area, the presence of *Amblyomma variegatum* and *Rhipicephalus microplus* has been reported but the competence of these ticks for RVFV transmission is unknown [35].

Multi-model inference allowed defining a “confidence set” of models according to which RVFV introduction occurred either in September or in October 2007, a stronger weight being attributed to the 1^st^ of these two dates, that corresponds to the end of the dry and cold season. This period corresponds to a rice transplant period in the study area, when rice fields are flooded for the first time since the previous rainy season. These conditions are consistent with the existence of an active mosquito population, even if the low temperatures are still unfavourable to mosquito proliferation. At that period of the year, breeders of the area need suitable cattle for labour in the rice fields. The money obtained from the sale of the previous harvest rice is available and breeders visit distant markets and buy renewal animals there. Field observations indicated that the closest cattle market is supplied by cattle of a breeding area which was heavily affected by RVFV in 2008 and 2009 and located in climatic condition favourable to competent RVFV mosquito vector [23]. According to the dealers of this market, the travel duration from the breeding area to the cattle market is about a week [23]. Considering that the RVFV viraemia and incubation period last about 4 days each, virus introduction from distant markets is possible. Nevertheless, field observations also showed that only a small number of cattle had been introduced from this breeding area in 2009. Virus introduction by trade from markets thus represents a real risk, but with a low occurrence probability. In Tanzania, cases of RVFV were reported from January to April 2007 [14]. Trade between Tanzania and Madagascar (mainly in Mahajunga harbour, in the north-west of the island) through the Comoros and Mayotte islands could have allowed vireamic cattle to be introduced. Afterwards, the favourable climatic conditions that prevail in the north of Madagascar (around Mahajunga harbour) could have allowed the RVFV to start spreading in the country.

The models selected in the confidence set accounted for a recurrent circulation of RVFV in the study area. However, this recurrent circulation is predicted to occur at a relatively low rate. This suggests a high probability of extinction of the transmission process, leading to a disappearance of RVFV from the study area. We used a deterministic implementation, that allowed using maximum likelihood for estimating parameters, likelihood being necessary to multi-model inference. In this deterministic implementation, infection dynamic events (e.g. contamination or immunization) and population dynamic events (e.g. calving or slaughter) were treated as continuous events, despite they could be considered as discrete events, especially in small populations (i.e. in villages with few cattle). More generally, deterministic approaches are less relevant for analyzing extinction probabilities than stochastic approaches [36]. Even if parameter estimation and model comparison is more complex (despite the recent advances obtained using Approximate Bayesian Computing methods [37]), a stochastic implementation of the model should allow analyzing the extinction probability of RVFV circulation in the study area [38], [39].

Several RVFV circulation modeling studies have been published. Most of them focus on theoretical issues such as the conditions of endemicity [40], or aim at predicting the potential impact of a RVFV introduction into disease-free countries such as Netherlands or US [41], [42]. The model proposed by Xue et al. 2012 [43] is devoted to South Africa where a large outbreak recently occurred [44]. Their network-based meta-population model was calibrated with observational data to reproduce the observed epizootic dynamics. However, this model did not take into account a potential direct transmission between cattle. Host interactions and movements are crucial for disease spread. In low-income countries, data on population density and flows are rare and, when available, most of the time coarse. The proposed model incorporates data on cattle population size and flows between villages, while being spatially explicit (villages and rice field locations). Previous modeling studies of RVFV circulation aimed at reproducing spatial epidemic in network [43], [45], [46] or at evaluating the involvement of mosquito vectors in RVFV spread [41] and persistence [40], under tropical or temperate climates [42]. They suggested the importance of the vector life span on the risk of onset of an outbreak and some of them allowed to build risk maps. Fischer et al. [42] also demonstrated the importance of a high vector-host ratios in the definition of the areas at risk. However, none of these previous modeling studies was truly spatially-explicit.

Spatialization allowed computing maps of predicted seroprevalence. These predicted seroprevalence levels appeared overestimated in the centre of the studied area, around the rice fields that were close to several villages. This could be explained by the fact that, in our model, mosquito are assumed to randomly bite cattle of villages located <1km of the rice field where their population is located. However, when seeking for a blood meal, a mosquito will probably rather be attracted by the nearest host, even if several villages are located within the mosquito flight distance. The spatialization method we used did not allow taking into account this clustering effect. This could have led to overestimate RVFV transmission in areas where rice fields are close to several villages.

In conclusion, our study suggests that, in Madagascar highlands, the recurrent circulation of RVFV is attributable both to vector-based transmission (for the main part), and to direct transmission at calving of viraemic cows. Socio-economic practices of this area appear determinant in virus introduction, spread and persistence. The seasonal coincidence of cattle introduction from markets and of the flooding of rice fields for rice transplant could allow RVFV to be introduced and start circulating locally some weeks before the beginning of the warm and wet season. Mosquito proliferation would then induce the epidemic peak. In the subsequent years, direct transmission at calving of viraemic cows, combined with vector-based transmission would support a recurrent circulation of RVFV. Finally, the traditional barter practice favours the contact between vectors and cattle when mosquito aggressivity is maximal (at twilight, around flooded rice field), hence the diffusion of the RVFV. The low level abundance of mosquito in the area may thus be compensated by socio-economic practices in relation with agricultural works using cattle for labour, allowing RVFV to be introduced, to spread and to persist for several years.

The selected models confirm that eco-climatic conditions prevailing in that area are not favourable to an endemic and high-level RVF transmission. However the commercial links existing between this area and regions where conditions are favourable (such as the south western part of the island) make it at risk for new introductions. Given the high value of cattle and the potential transmission to human, a passive surveillance system would be relevant to allow implementing early information and prevention measures and decreasing the health and economic impact of the disease in case of a new outbreak.

As Jeanmaire et al. [47] emphasized, and despite a rather temperate climate the virus intensively circulated in most of highland districts in 2008, suggesting its ability to emerge in other temperate parts of the world given an introduction. The proposed model could be used (i) to analyze the outbreaks risk in similar areas given local conditions (trade, vectors species, land use) (ii) to evaluate the efficiency of control measures such as vaccination campaigns (iii) and to evaluate the potential economic impacts of a RVFV outbreak in this area of a low-income country where economic condition and survival is related to rice crop for which cattle are essential.

## Supporting Information

Text S1 Model structure.
(DOCX)
