# Supplementary material for: A Spatially Explicit Metapopulation Model and Cattle Trade Analysis Suggests Key Determinants for the Recurrent Circulation of Rift Valley Fever Virus in a Pilot Area of Madagascar Highlands
Source: PLoS Negl Trop Dis. 2014 Dec 4;8(12):e3346. doi: 10.1371/journal.pntd.0003346 (PMC4256285; doi:10.1371/journal.pntd.0003346)
Supplement: Text S1 — Model structure. (DOCX) [file pntd.0003346.s001.docx]

***Model***

*Cattle populations*

Four health states were distinguished for cattle: susceptible (*S*), incubating (*E*), viraemic (*I*) and immune (*R*). Eight state variables were associated with each village, of the form $H_{x}^{g}\left(t\right)$, where $H\in\left\{S, E,I,R\right\}$ is a health state, *x* ($1\leq x\leq X$) is a village number (*X* being the total number of villages), and $g\in\left\{T, B\right\}$ denotes a group of breeders: those who use usual trade only (*T*), and those who use cattle barter in addition to usual trade (*B*). For example, $S_{x}^{T}\left(t\right)$ denoted, at day *t*, the number of cattle in the S state, that belong to a breeder of village *x* who does not practice animal barter but only usual trade.

The dynamic of the population of cattle living in village *x* and belonging to a breeder of group *g* was defined by the following equations (1)-(4):

$$S_{x}^{g}\left(t+1\right)=S_{x}^{g}\left(t\right)\left[1-\mu\right]+\nu N_{x}^{g}\left(t\right)-\lambda_{x}^{g}\left(t\right)\left[1-\mu\right]S_{x}^{g}\left(t\right)$$

$$E_{x}^{g}\left(t+1\right)=E_{x}^{g}\left(t\right)\left[1-\mu\right]\left[1-\psi^{-1}\right]+\lambda_{x}^{g}\left(t\right)\left[1-\mu\right]S_{x}^{g}\left(t\right)$$

$$I_{x}^{g}\left(t+1\right)=I_{x}^{g}\left(t\right)\left[1-\mu\right]\left[1-\rho^{-1}\right]+\psi^{-1}\left[1-\mu\right]E_{x}^{g}\left(t\right)$$

$$R_{x}^{g}\left(t+1\right)=R_{x}^{g}\left(t\right)\left[1-\mu\right]+\rho^{-1}\left[1-\mu\right]I_{x}^{g}\left(t\right)$$

where:

- $N_{x}^{g}$is the total number of cattle belonging to a breeder of group *g* in village *x* ($N_{x}^{g}=S_{x}^{g}+E_{x}^{g}+I_{x}^{g}+R_{x}^{g}$),
- *μ* is the daily mortality rate,
- *ν* is the daily per-capita birth rate.
- $\lambda_{x}^{g}\left(t\right)$ is the daily probability of infection for cattle belonging to breeders of group *g* in village *x*,
- *ψ* is the duration of the incubation period,
- *ρ* is the duration of viraemia.

The daily probability of infection for cattle belonging to breeders that do not practice cattle barter but only usual trade was:

$$\lambda_{x}^{T}\left(t\right)=1-exp\left(\lambda_{x}^{1}\left(t\right)+\lambda_{x}^{2}\left(t\right)\right)$$

where:

- $\lambda_{x}^{1}\left(t\right)$ is the force of infection due to direct transmission, and
- $\lambda_{x}^{2}\left(t\right)$is the force of infection due to vector-based transmission, in village *v* at day *t*.

The force of infection due to direct transmission, in village *v* at day *t*, was proportional to the proportion of viraemic cows that calve during day *t*:

$$\lambda_{x}^{1}\left(t\right)=\beta\nu\frac{I_{x}^{B}\left(t\right)+I_{x}^{T}\left(t\right)}{N_{x}^{B}\left(t\right)+N_{x}^{T}\left(t\right)}$$

where *β* is the direct transmission coefficient.

The size of the vector population of a given rice field *y* was assumed proportional to its area *A_y_*. The daily variations of this population size were forced by the relative abundance of vectors at day *t* (denoted $\alpha\left(t\right)$, with $0\leq\alpha\left(t\right)\leq1$). The number of mosquito per host in rice field *y* at day *t* was then: $H_{v} \alpha\left(t\right) A_{y}$ , where *H_v_* is the vector/host ratio. This allowed defining the force of infection due to vector-based transmission, in village *x* at day *t*:

$$\lambda_{x}^{2}\left(t\right)=\sum_{y\in R\left(x\right)} \text{γ}^{-1}\left[H_{v} \alpha\left(t\right) A_{y}\right]I_{y}\left(t\right)$$

where:

- $R\left(x\right)$ is the set or rice fields located in the neighborhood of village *x*,
- *γ* is the duration of the gonotrophic cycle period ($\gamma^{-1}$being then the proportion of mosquitoes that take a blood meal at day *t*),
- *H_v_* is the vector/host ratio, considering the mosquito production by one surface unit of a rice field (*i.e.* 1m^2^)
- *A_y_* is the area of rice field *y*,
- *α*(*t*) is the relative abundance of mosquitoes at day t,
- $I_{y}\left(t\right)$is the proportion of infectious vectors in rice field *y* at day *t*.

The daily probability of infection for cattle belonging to breeders that do practice cattle barter (in addition to usual trade) took into account the fact that part of their exposure to mosquito bites occurred near the villages where they had been brought by the breeder for barter:

$$\lambda_{x}^{B}\left(t\right)=1-exp\left(\lambda_{x}^{1}\left(t\right)+\left(1-p_{B}\right)\lambda_{x}^{2}\left(t\right)+p_{B}\lambda_{x}^{3}\left(t\right)\right)$$

where:

- $p_{B}$ is the proportion of cattle exposure to mosquito bites that occur near the villages with which the breeder used to barter animals, and
- $\lambda_{x}^{3}\left(t\right)$ is the average force of infection due to vector-based transmission, at day *t*, in the villages where animals are bartered by breeders of village *x*: $\lambda_{x}^{3}\left(t\right)=\frac{1}{B\left(x\right)}\sum_{i\in B\left(x\right)} \lambda_{i}^{2}\left(t\right)$

where *B*(*x*) is the set of villages where breeders of village *x* usually go to barter animals, and $B\left(x\right)$ denotes the size of this set.

Animal movements (either bartered or sold) were assumed instantaneous and were computed as follows, for each health state *H* and for each breeder group *g*:

$$H_{x}^{g}\left(t\right)=H_{x}^{g}\left(t\right)+\left[\sum_{1\leq i\leq X} \text{Ω}_{\text{g}}\left(i, x\right)\frac{H_{i}^{g}\left(t\right)}{N_{i}^{T}\left(t\right)+N_{i}^{B}\left(t\right)}-\text{Ω}_{\text{g}}\left(x, i\right)\frac{H_{x}^{g}\left(t\right)}{N_{x}^{T}\left(t\right)+N_{x}^{B}\left(t\right)}\right]$$

where:

- *X* is the number of villages,
- $\text{Ω}_{\text{g}}\left(i, j\right)$ denotes the average number of animals moved daily from village *i* to village *j*, for breeders of group *g*,
- $N_{x}^{T}\left(t\right)$and$N_{x}^{B}\left(t\right)$ are the total numbers of animals living in village *x* at day *t*, that belong to breeders of group T (usual trade without animal barter) and B (usual trade and animal barter), respectively.

*Mosquito populations*

The mosquito population dynamic (oviposition, larval development, emergence) is forced by two time-varying parameters: the daily abundance of mosquitoes (denoted $\alpha\left(t\right)$, with $0\leq\alpha\left(t\right)\leq1$, see above), and the parous rate (denoted $\eta\left(t\right)$, with $0\leq\eta\left(t\right)\leq1$, the proportion of female mosquitoes that have laid eggs at least once). The value of both parameters varied daily.

Four state variables were associated with each rice field *y*: $N_{y}\left(t\right)$, $S_{y}\left(t\right)$, $E_{y}\left(t\right)$, and $I_{y}\left(t\right)$ that respectively denoted the proportion of mosquitoes, at day t, in the health state *N*, *S*, *E* and *I*. The dynamic of the mosquito population of rice field *y* was defined by the difference equations (10)-(13):

$$N_{y}\left(t+1\right)=1-\eta\left(t+1\right)$$

$$S_{y}\left(t+1\right)=\frac{\eta\left(t+1\right)}{1-N_{y}\left(t\right)\left[1-\gamma^{-1}\right]}\left[S_{y}\left(t\right)\left[1-\gamma^{-1}\lambda_{y}\left(t\right)\right]+\gamma^{-1}\left[1-\lambda_{y}\left(t\right)\right]N_{y}\left(t\right)\right]$$

where:

- $\eta\left(\text{t}\right)$ is the parous rate a day *t* (i.e. the proportion of mosquitoes that have taken at least one blood meal since emergence),
- $\gamma$ is the duration of the gonotrophic period ($\gamma^{-1}$ being then the proportion of mosquitoes that take a blood meal),
- $\lambda_{y}\left(t\right)$ is the force of infection for mosquitoes of rice field *y* that take a blood meal at day *t*, and
- is the duration of the extrinsic incubation period.

The transitions from state E to state I (that represents the end of the extrinsic incubation period) was thus assumed to occur at a constant rate (), whereas the transition rate from *N* and *S* to *E* (that represents the infection of vectors) was the product of the proportion of mosquitoes that take a blood meal ($\gamma^{-1})$ by the force of infection to which these mosquitoes were exposed ($\lambda_{y}\left(t\right)$). The latter was the proportion of viraemic cattle among those that may be bitten by the mosquitoes of the rice field *r*, either because they live in the neighboring villages, or because they have been brought there by a breeder that wants to barter some of his animals:

$$\lambda_{y}\left(t\right)=\sum_{i\in V\left(y\right)} \frac{I_{i}^{T}\left(t\right)+I_{i}^{B}\left(t\right)\left[1-p_{B}\right]+\frac{1}{B^{-1}\left(i\right)}\sum_{j\in B^{-1}\left(i\right)} p_{B}I_{j}^{B}\left(t\right)}{N_{i}^{T}\left(t\right)+N_{i}^{B}\left(t\right)\left[1-p_{B}\right]+\frac{1}{B^{-1}\left(i\right)}\sum_{j\in B^{-1}\left(i\right)} p_{B}N_{j}^{B}\left(t\right)}$$

where:

- *V*(*y*) is the set of villages located in the neighborhood of rice field *y*,
- $B^{-1}\left(x\right)$is the set of villages from where originate the breeders that propose barters to breeders of village *x* (*i.e.* the reciprocal of $B\left(x\right)$).
